# Counselling practices in community pharmacies in Riyadh, Saudi Arabia: a cross-sectional study

**DOI:** 10.1186/s12913-015-1220-6

**Published:** 2015-12-15

**Authors:** Sinaa Alaqeel, Norah O. Abanmy

**Affiliations:** Clinical Pharmacy Department, College of Pharmacy, King Saud University, Riyadh Kingdom of Saudi Arabia, PO Box 376316, Riyadh, 11335 Kingdom of Saudi Arabia

**Keywords:** Community pharmacy, Patient simulation, Counselling, Lay perspectives, Patient safety

## Abstract

**Background:**

Community pharmacists play a crucial role in optimising medication use and improving patient outcomes, whilst preventing medication misuse and reducing costs. Evidence suggests that pharmacists counselling improves clinical outcomes, quality of life, drug and disease knowledge and reduces health service utilisation. This study aims to investigate the counselling practices of community pharmacists in Riyadh, the capital of Saudi Arabia.

**Methods:**

The study consisted of two parts: simulated patients (SPs) visits to observe actual counselling practices, and a cross-sectional survey of community pharmacists to assess their reported counselling practices. In the SPs method, there were four scenarios involving four medications. Scenarios 1 and 2 concerned drug–drug interactions, scenario 3 concerned the proper time of administration, and scenario 4 concerned side effects. The simulated visits were conducted between April and May 2012. A four-sections questionnaire was distributed in the same period.

**Results:**

We conducted 161 simulated visits. Out of the 161 visits a medicine was dispensed in 150 visits. When SPs requested medications, pharmacists asked questions during 15 visits (10.0 %), provided information during 7 visits (4.6 %), and both asked questions and provided information, i.e. provided counselling, during 4 visits (2.6 %). When the SPs started to be inquisitive and demanded information, pharmacists asked SPs questions during 71 visits (47.3 %), provided information during 150 visits (100 %), and both asked questions and provided information, i.e. provided counselling, during 65 visits (43.3 %). Information regarding dose was the most common type of information provided in 146 visits (97.3 %). After the SPs started to be inquisitive and probed for information, only 10 % were counselled on precautions. In the cross-sectional survey, four hundred pharmacists were approached and 350 agreed to participate in the questionnaire (87 % response rate). Of the respondents, 223 (63.7 %) reported that they usually or always tell the patient about the purpose of medicines or the diagnosis, 302 (86.2 %) reported that they usually or always give patient information on how to use or apply the medicine; 299 (85.3 %) said they were satisfied with their counselling practices.

**Conclusions:**

The present study highlights the current deficiencies in appropriate dispensing practices and medication counselling at community pharmacies in Saudi Arabia. Policy makers, stakeholders, and researchers should collaborate to design interventions to improve the current dispensing practices at community pharmacies across the country.

**Electronic supplementary material:**

The online version of this article (doi:10.1186/s12913-015-1220-6) contains supplementary material, which is available to authorized users.

## Background

Community pharmacists play a crucial role in optimising medication use and improving patient outcomes, whilst preventing medication misuse and reducing costs [1, 2]. Patient counselling is an important service provided by community pharmacies.

Patient education and counselling usually occur at the time prescriptions are dispensed but may also be provided as a separate service [3]. A systematic review findings indicate that pharmacists led counselling improves clinical outcomes, quality of life, drug and disease knowledge, patients’ satisfaction with service, and economic outcomes [4]. It is worth noting that the counselling was more comprehensive and extended beyond time of dispensing a medication in the majority of the included trials in the review. At the time of dispensing, evidence exist to suggest that community pharmacists’ interventions such as counselling encourage appropriate medicine use and prevents drug related problems [5, 6].

Many professional organizations have published guidelines that provide recommendations to pharmacists on how to educate and counsel patients on both prescription and non-prescription medicines [3, 7]. Although the scope of content of the counselling recommended in each guideline varies, all agree on providing the following information: name and description of the medicine, indications, route of administration, dose and dosage form, directions for use, duration of therapy, special directions, precautions, side effects, and contraindications [3, 7]. All guidelines also emphasize that pharmacists need to ask a series of questions to identify a patient’s understanding of their medications, and to meet the specific needs of each patient and/or caregiver [3, 7].

There are approximately 7,322 community pharmacies in Saudi Arabia staffed by an estimated 12,506 pharmacists [8]. Community pharmacies are privately owned and there are numerous chains of pharmacies. Pharmacies are located in a variety of premises, with the majority in main streets and some linked with private healthcare clinics. According to the law regulating Saudi pharmacy practice [9], all professional operations in a pharmacy must be performed by a licensed pharmacist. Passing the Saudi Commission for Health Specialties licensing exam is one requirement for the licensing of pharmacists. The law prohibits medication dispensing without a prescription, unless the medication is designated as over-the-counter (OTC). The law also mandate that medications purchased from a community pharmacy are dispensed in their original packages with information package insert. The outer package must include information such as the name of the medication, active ingredients, pharmaceutical form, strength, storage condition, price, and manufacturer.

A Saudi survey of 500 patients attending primary healthcare centres indicated that 35.4 % of respondents had practiced self-medication [10]. The authors defined this as the use of drugs to treat self-diagnosed disorders or symptoms, or the intermittent or continued use of a prescribed drug for chronic or recurrent disease or symptoms, in the preceding 2 weeks. The inappropriate use of medicines by self-medicating consumers is reported to be a factor contributing to medication errors in the community, either alone or in combination with other factors [11]. Community pharmacists play a key role in counselling self-medicated patients. They monitor the use of non-prescription medicines, identify drug-related problems, and intervene when necessary to ensure that patients use medicines safely, appropriately, and effectively [2, 5]. In Saudi Arabia, it is common for prescription only medicines (POM) to be supplied by community pharmacists without a doctor’s prescription [12, 13]. This means that community pharmacists are often the only point of contact for patients before initiating drug therapy, not only for OTC medication but also for POM.

This study aims to investigate the counselling practices of community pharmacists in Riyadh, the capital of Saudi Arabia when dispensing OTC and POM without a prescription. Using simulated patients (SPs) visits and a questionnaire, we assessed the following parameters: counselling rate, types of questions asked, and information provided during the counselling process.

## Methods

The study consisted of two parts: simulated patients visits and a cross-sectional survey of community pharmacists. The simulated patients visits were used to assess ‘real’ (unrehearsed) counselling practices, while the survey was used to assess the community pharmacists’ reported counselling practices.

### The simulated patient method

The simulated patient (SP) method was used to determine how community pharmacists currently provide patient counselling. This method has been employed extensively in pharmacy practice-based research [12–18]. Within the pharmacy context, a simulated patient is ‘an individual who is trained to visit a pharmacy to enact a scenario that tests a specific behaviour of the pharmacist or pharmacy staff [19].

#### The simulated patients

The SPs were four female pharmacy students. Students have been used as simulated clients in previous research [12, 14, 16, 17]. We used four students, as a systematic review of SP research in pharmacy practice recommends using a minimum of two SPs [19]. The participation of the students was voluntary. The authors trained the SPs using role-play to ensure the scenario was performed in a consistent manner. Repeated rehearsal ensured reliability of the simulated scenario. The actors used lay language and refrained from using any jargon.

#### The pharmacies

The authors stratified Riyadh city using geographical areas: north, south, west, east, and central. A convenient sample of pharmacies in each area was selected. Each pharmacy was visited once by one of the SPs. A pilot study (*N* = 8 pharmacies) to test the content and delivery of the scenarios was conducted. The results of the pilot study were reviewed by the authors to ensure consistency in the collection of data by the SPs. The SPs visits for the current study were conducted between April and May 2012.

#### The scenarios

There were four scenarios (Table 1). Two of the medications used in our scenarios are classified as POM in scenario 2 (Amoxil) and scenario 4 (Zocor).

**Table 1:** Descriptions of scenarios

***Scenario 1***	***Scenario 2***
SP enters the pharmacy and asks: “May I have Ferose?”	SP enters the pharmacy and asks: “May I have Amoxil?”
If asked, the SP told the pharmacy staff that she has not previously taken the medicine, that it is for personal use, that she is 20 years old, and that she has anaemia and sometimes takes antacid for stomach upset. She also said that she has not received any information from her doctor.	If asked, the SP told the pharmacy staff that she has not previously taken the medicine, that it is for personal use, that she is 20 years old, and that she has sore throat and is on birth control pills (Genera). She also said that she has not received any information from her doctor.
If pharmacists provided no counselling, SPs asked the following:	If pharmacists provided no counselling, SPs asked the following:
- May I take Ferose at any time?	- May I take Amoxil at any time?
- May I take Ferose before or after a meal?	- May I take Amoxil before or after a meal?
- I am using antacids occasionally. Is it OK to take both antacid and Ferose at the same time?	- I am on Genera. Is it OK to take both Amoxil and Genera at the same time?
***Scenario 3***	***Scenario 4***
SP enters the pharmacy and asks: “May I have Moxal?”	SP enters the pharmacy and asks: “May I have Zocor?”
If asked, the SP told the pharmacy staff that she has not previously taken the medicine, that it is for personal use, that she is 20 years old, and that she has kidney failure. She also said that she has not received any information from her doctor.	If asked, the SP told the pharmacy staff that the medicine is for her mother, who is 70 years old, has not previously taken the medicine, and has high cholesterol. She also said that she has not received any information from her doctor.
If pharmacists provided no counselling, SPs asked the following:	If pharmacists provided no counselling, SPs asked the following:
- May I take Moxal at any time?	- Are there any side effects of this medicine that I should watch for?
- May I take Moxal before or after a meal?	

In scenario one, the patient is anaemic and asking for iron (Ferose^R^). However, she is also frequently taking antacid, which could prevent full iron absorption and delay the response to iron therapy [20]. In scenario two, the patient is asking for amoxicillin (Amoxil^R^), which is a penicillin antibiotic known to alter intestinal flora that, in turn, reduces the enterohepatic circulation of estrogen metabolites. Concomitant use with birth control pills is associated with unintended pregnancies and menstrual changes. Therefore, pharmacists are expected to provide SPs with a caution of unplanned pregnancy as a possible result of the interaction between the antibiotic and contraceptive pills [20]. In scenario three, a patient with renal failure is asking for the antacid Moxal^R^ (an aluminium-containing antacid) as a phosphate-binding agent. Pharmacists are expected to stress the need to time the administration of phosphate-binding agents with food intake to prevent phosphate absorption that leads to hyperphosphatemia in patients with renal failure [20]. In scenario four, SPs request simvastatin (Zocor^R^), which is a lipid-lowering agent known to induce myopathy and rhabdomyolysis. Pharmacists are expected to provide the SPs with information regarding potential side effects and ask them to report unexplained muscle pain, tenderness, weakness, and dark-coloured urine [20].

SPs were given clear instructions to not provide further information unless requested by the pharmacy staff member. If requested, the information subsequently provided is presented in Table 1. If pharmacists dispensed medications without providing any counselling, the SPs were instructed to pose specific questions to draw the pharmacist’s attention to the counselling points that need to be addressed (Table 1).

#### Documentation of the counselling process

To minimize recall bias, outside the pharmacy the counselling process was documented on an assessment form immediately after each visit. The form (available from the corresponding author) was designed based on forms from previously published work [17, 18]. The SPs were familiarised with the standardised data collection form before initiating the visits. They also practiced using the form during the pilot study.

In all the scenarios, pharmacists were assessed on whether they provided counselling, using the same categorisation as used by Tully et al. [18]. “No questioning” was when the SP was not asked about previous use, use of other medicines, allergies, and concerns about using this medicine. “No information provision” was when no verbal information was given about name of the medicine, dose, how to take the medication, duration of use, and possible adverse drug. “No counselling” was a lack of both questioning and information provision. Furthermore, for each scenario pharmacists were expected to provide any special directions or precautions.

### Cross sectional survey of community pharmacists

The questionnaire in this study was a slightly modified form of the questionnaire used in a previous study assessing dispensing practices in Cyprus [17]. Two local pharmacists checked the questionnaire for interpretation issues and suitability to the Saudi setting.

The final questionnaire consisted of four sections (see Additional file 1). The first section requested information about the pharmacists’ demographics, average dispensing time, and number of patients waiting. The second section comprised 11 statements regarding information that could be provided for customers during the dispensing process. The pharmacist answered by providing an estimate of how often they offered this information to patients. In the third section, the pharmacists’ views about their counselling were assessed. The final section investigated any barriers to counselling.

As with the SPs study component, we stratified Riyadh city by geographical areas and a convenient sample of pharmacies from different areas was selected. Pharmacies were visited by volunteer pharmacy students who asked the pharmacists if they willing to participate in a study about patient counselling in community pharmacies. Those who agreed to participate were provided with a hard copy of the questionnaire, which they completed while the students were waiting in the pharmacy. The questionnaires were not distributed to the same pharmacies that were visited by SPs. The questionnaires were distributed during different days of the week at different times of the day to avoid targeting pharmacists only during busy hours or days. The survey period was between April and May 2012.

### Data analysis

Data from the SPs visits and survey were entered by a research assistant and checked by one of the authors (NA). The data were stored and analysed using Excel 2003. Continuous data are reported as mean and standard deviation (SD). Categorical data are expressed as frequencies and percentages.

### Ethical approval

This study involves observation of pharmacists’ behaviour and does not interfere with patient care. The study was reviewed and approved by The institutional Review Board at king Saud University. Information obtained is recorded in such a manner that pharmacists involved cannot be identified and results are reported in an anonymous way. This ensures that participating pharmacists are not at any risk of criminal or civil liability, and it does not damage their employability or reputation.

## Results

### The simulated patients (SPs) method

One hundred and sixty one visits were conducted: Scenario 1 = 49 (30 %), Scenario 2 = 50 (31 %), Scenario 3 = 20 (12 %), Scenario 4 = 42 (21 %). Two of the medications used in our scenarios are classified as POM. Nonetheless, pharmacists dispensed Amoxil without asking for a prescription in all 50 SPs visits and dispensed Zocor without a prescription in 31 out of 42 SPs visits (74 %). This means out of the 161 visits a medicine was dispensed in 150 visits.

The SPs had to wait on 65 out of 161 visits (40 %). Time spent waiting for a visit was less than 1 min on 30 visits (18.6 %), 1–5 min on 30 visits (18.6 %), and more than 6 min on 5 visits (3.1 %). On 67 occasions (41.6 %), there were no other customers waiting. During other visits, there were 1–2 customers waiting on 57 occasions (35.4 %) and 3–5 customers waiting on 16 occasions (9.9 %). These values do not total 161 where there is missing data.

Data regarding the presence and content of counselling during dispensing are reported in Table 2. When SPs requested medications, pharmacists asked questions during 15 visits (10.0 %), provided information during 7 visits (4.6 %), and both asked questions and provided information, i.e. provided counselling, during 4 visits (2.6 %).

**Table 2:** Description of counselling received by simulated patients requesting medication in the four scenarios

	Total ^a,b^ *n* = 150(%)	Scenario 1 *n* = 49	Scenario 2 *n* = 50	Scenario 3 *n* = 20	Scenario 4 *n* = 31
Counselling before SP demanded information					
Asked questions	15(10.0)	1	1	0	13
Provided information	7(4.6)	1	2	0	4
Counselling after demanding information^a^					
Asked questions	71(47.3)	18	11	14	28
Provided information	150(100.0)	48	50	20	32
Questions asked about^a^					
Who is medicine for	24(16.0)	2	4	3	15
Whether had taken this medicine before	12(8.0)	0	0	0	12
If other medicines currently being taken	14(9.3)	1	0	0	13
If allergic to any medicine	11(7.3)	0	0	0	11
If have any questions or concerns about this medicine	4(2.6)	1	0	0	3
Information provided about					
Name of the medicine	10(6.6)	7	1	0	2
Dose	146(97.3)	46	50	19	31
How to take the medication (e.g. before or after meal)	109(72.7)	34	45	20	10
Duration of use	20(13.3)	3	5	3	9
Possible adverse drug reactions, warnings, and precautions	15(10.0)	3	2	2	8

^a^The total number sometimes exceeds the number of visits as pharmacists may ask more than one question or provide more than one type of information during a visit

^b^Out of the 161 visits a medicine was dispensed in only 150 visits

When SPs started to be inquisitive and demanded information, this prompted pharmacists to ask questions during 71 visits (47.3 %), provide information during 150 visits (100 %), and both ask questions and provide information, i.e. provide counselling, during 65 visits (43.3 %). The types of questions asked and information given are reported in Table 2.

The majority of pharmacists did not inquire about previous use of requested medications, concomitant drugs used, or any history of drug allergy. Information on dose was the most common type of information provided during 146 visits (97.3 %). After the SPs started to be inquisitive and probed for information, only 10 % were counselled on precautions.

### Cross-sectional survey of community pharmacists

Four hundred pharmacists were approached and 350 agreed to participate in the questionnaire (87 % response rate). Demographic information of the respondents is provided in Table 3. The majority of respondents were under 40 years of age (*n* = 321, 91.7 %). Only 15 respondents (4.2 %) were of Saudi nationality and the remaining were expatriates. Only 247 respondents provided their nationality and the majority were Egyptian (*n* = 212, 60.5 %). The average experience as a community pharmacist was 5 years (SD 4.5) inside Saudi and 3 years (SD 2.4) outside Saudi.

**Table 3:** Sociodemographic characteristics of respondents (*n* = 350)

	Number (%)^a^
Age	
20–30	192 (54.8)
31–40	129(37.1)
41–50	23(6.5)
> 50	6(1.6)
Nationality	
Saudi	15(4.2)
Non-Saudi	333(95.1)
Degree	
Bachelor	268(76.5)
Doctor of pharmacy	72(21.5)
Others	3(0.85)
Years working as a community pharmacist	
Inside Saudi Arabia	5 (SD 4.5, min <1 year, max 30 years)
Outside Saudi Arabia	3 (SD 2.4, min <1 year, max 16 years)

^a^The total number does not always add up to 350 due to missing data

Table 4 illustrates the information that pharmacists claimed to provide to clients. Respondents claim that they frequently inform the patients about the dosing and the instructions for taking drugs, and whether to take medication with food or on an empty stomach. According to respondents, information on potential side effects, drug interactions, and food interactions is provided less frequently to patients.

**Table 4:** Statements pharmacists identified as information they provide to patients about their prescription/medications (*n* = 350)^a^

	Always (%)	Usually (%)	Often (%)	Sometimes (%)	Never (%)
The purpose of medication or diagnosis	126(36.0)	97(27.7)	64(18.3)	53(15.1)	4(1.1)
Dosing of the drugs	249(71.1)	72(20.5)	22(6.2)	4(1.1)	0(0)
Information on how to use the medication and its application	221(63.1)	81(23.1)	26(7.4)	8(2.3)	1(0.28)
Medication to be taken with food or on an empty stomach	213(60.8)	85(24.3)	25(7.1)	15(4.3)	3(0.85)
Duration of use	164(46.8)	73(20.8)	65(18.5)	31(8.8)	4(1.1)
Possible side effects	26(7.4)	33(9.4)	53(15.1)	76(21.7)	13(3.7)
Drug interactions	47(13.4)	56(21.1)	74(21.1)	139(39.7)	31(8.8)
Food interactions	55(15.7)	79(22.5)	81(23.1)	105(30.0)	23(6.5)
Importance of compliance	69(19.7)	68(22.5)	95(27.1)	84(24.0)	18(5.1)
Storage conditions	91(26.6)	79(22.5)	70(21.0)	78(22.2)	23(6.5)
Availability of generic medication	57(16.2)	84(24.0)	86(25.6)	93(27.6)	29(8.2)

^a^The total number does not always add up to 350 because of missing data

The majority of pharmacists (86.6 %) believe that patients feel comfortable to consult their pharmacists about their medication/medical condition (Table 5). When asked about barriers to counselling (Table 6), over half of respondents indicated that pharmacists are too busy (59.6 %) and a similar proportion reported that pharmacists do not have the patient medical history (61.9 %).

**Table 5:** Statements given by pharmacists regarding their counselling practice (*n* = 350)^a^

	Strongly agree (%)	Agree (%)	Unsure (%)	Disagree (%)	Strongly disagree (%)
Patients are comfortable in consulting me about their medication/medical condition	106(30.3)	197(56.3)	33(9.4)	12(3.4)	1(0.28)
I use all opportunities to clarify patients’ understanding of my counselling	118(33.7)	172(49.1)	48(13.7)	8(2.2)	1(0.28)
Patients understand the information I provide them	87(24.8)	172(49.1)	68(19.4)	13(3.7)	3(0.85)
I confirm and clarify the understanding of the patient	102(29.1)	172(49.1)	58(16.5)	6(1.7)	2(0.57)
I am satisfied with my counselling practice	130(37.1)	169(48.2)	34(10)	9(2.6)	4(1.1)

^a^The total number does not always add up to 350 because of missing data

**Table 6:** Statements given by pharmacists regarding barriers to counselling (*n* = 350)^a^

	Strongly agree (%)	Agree (%)	Unsure (%)	Disagree (%)	Strongly disagree (%)
Pharmacists have limited drug information resources	41(11.7)	94(26.8)	48(13.7)	101(28.8)	64(18.3)
Pharmacists are too busy	68(19.4)	141(40.2)	51(14.5)	67(19.1)	18(5.1)
Pharmacists do not have the patient medical history	75(21.4)	142(40.5)	69(19.7)	40(11.4)	19(5.4)
Pharmacists lack confidence in their knowledge	27(7.7)	60(17.1)	50(14.2)	107(30.5)	105(30.0)

^a^The total number does not always add up to 350 because of missing data

## Discussion

The present study evaluated the counselling practices of community pharmacists in Riyadh, Saudi Arabia. Results obtained from the visits using SPs were compared with those obtained in the survey, revealing important discrepancies regarding the frequency of information provided to customers. In the survey, the majority of the respondents claimed that they always provide information on dose, duration of use, and how to use the medication. Nevertheless, actual dispensing practices showed that the majority of SPs were informed about such information only when they started to be inquisitive and probed for information. Others have reported similar inconsistencies between self-reported behaviour of pharmacists in interviews and their actual dispensing practice measured using SPs [17]. It is possible that pharmacists provide more socially desirable responses in the questionnaires. Another possible explanation is that the survey responses may refer to general counselling practice, while the SPs component reflects counselling practice on specific occasions when patients request a medicine by name. Evidence suggests that the latter involves less counselling [15, 21, 22].

Counselling rates reported in international literature vary from 8 to 100 %, depending on the research methods used [7]. The rate of counselling observed in this study is very low (3 %). However, it improved to 43 % when SPs were inquisitive and requested more information. This improvement in observed counselling rate is consistent with previous research reporting an association between patient question-asking behaviour and the provision of information [21, 22]. This improvement in pharmacist-patient communication demonstrates the importance of finding ways to encourage patients to ask questions to community pharmacists.

When dispensing, the majority of pharmacists did not inquire about previous use of the requested medications, concomitant drugs, or history of drug allergy. In a study involving simulated patients visiting community pharmacies in Riyadh with symptoms of specific clinical illnesses, antibiotics were dispensed without inquiring about associated symptoms or history of drug allergy, and only 23 % of pharmacists inquired about pregnancy status [12]. Other studies also report that pharmacists’ assessment of symptoms and questioning concerning medication history are inadequate [15–18, 21, 22]. One explanation for this finding could be that our SPs were asking for specific products. In the context of non-prescription medicines, research suggests that product-based requests result in less information being asked or information elicited by pharmacists than do symptom-based [15, 21, 22]. Another plausible explanation is that consumers might expect to make an OTC purchase without being questioned [23]. However, in this study two medications were POM. For one of these, Amoxil, there was no assessment or request for a prescription. Pharmacists’ assessment with the other, Zocor, was a little better, but still unsatisfactory.

Information regarding dose was the most common type of information provided (97 % of the SPs visits), while a very small proportion of SPs were counselled on precautions. This is consistent with findings in previous research reporting that information on precautions, side effects, interactions, contraindications, and storage is less likely to be given by community pharmacists [7, 12, 13, 17, 18]. Interestingly, in our survey approximately half of respondents reported that they sometimes or never counselled on side effects and drug interactions. Pharmacists may think that too much information could deter patients from taking their medications [24]. However, research suggests that patients want specific information about side effects, duration of treatment, and the range of available treatment options [24].

Although two of the medications used in the scenarios are POM, pharmacists requested the prescription on only 11 out of 92 SPs visits. This confirms findings from previous research that POMs, such as antibiotics, antihypertensives, and antipsychotics could be obtained easily without a medical prescription in Saudi Arabia [12, 13]. A Saudi study asked pharmacists (*n* = 60) for the reasons they violated the law and dispensed POM without a prescription [13]. The most common reasons given were that pharmacists do not know the prescription status (i.e. POM or OTC) of many medications, patients ask for medications by name, and patients can only afford the pharmacy visit. Some also reported that ‘if we did not sell it somebody else will’ [13]. These results suggest that the reasons for such malpractice are multifactorial, and multiple approaches are required to correct it.

### Implications for policy and research

The intensity of regulatory mechanisms has been proposed as a factor influencing counselling practice [25]. The Saudi Executives Roles for Institutional and Pharmaceutical Products Law [9] provide no clear regulation on what is expected of community pharmacists during the dispensing of medications. Therefore, the development of legislation or guidelines setting out national standards that clearly stipulate what is expected of community pharmacists during the counselling process is needed. Such guidelines should be supplemented by appropriate strategies for dissemination and implementation.

There should be stringent enforcement of the national regulations that require a valid prescription to dispense a POM in community pharmacies. The Saudi Ministry of Health have published a frequently updated version of the Saudi OTC-Directory since 2000. Additionally, an electronic list of medications licensed in Saudi Arabia, including their prescription status, is available on the Saudi FDA website [26]. However, this seems inadequate and relevant agencies should develop strategies to ensure efficient dissemination of information about the legal status of medications to all community pharmacies. Furthermore, the laws and regulations of the Saudi healthcare system should be part of Saudi Commission for Health Specialties licensing exam for pharmacists. This is especially important given that most community pharmacists in Saudi Arabia are expatriates.

Evidence for effective strategies to improve counselling practices is limited. However, some interventions show promising results, including continuous long-term postgraduate education [27] and simulated patient visits to assess the current practice followed by feedback [16, 21]. To promote longer-term changes in counselling practices, systematic action is required, coordinated between relevant stakeholders and at different levels. Pharmacy owners and pharmacists will need support and resources to improve existing practice. These include management systems, medicine information systems and databases, and up-to-date basic and continuing education. At the individual pharmacist level, competencies should be updated to meet patient-centred practice requirements.

In this study, we only considered the provision of counselling and not the quality of counselling. Therefore, future studies that examine appropriateness and quality of counselling practices are needed. Future research should also investigate further the factors that hinder community pharmacists from counselling patients. These factors may relate either to the community pharmacists themselves, for example competencies and willingness, or to organizational factors, for example regulations and reimbursements.

### Limitations of the study

Some limitations of our study are outlined below. Our results should be generalised with caution to the general population due to our use of convenience sampling, as this method can lead to the under-representation or over-representation of particular groups within the sample. Further limitations include the use of students as simulated patients, who may not have been as convincing or as practiced as paid actors. Furthermore, the SPs were all young females, and generalization of the findings to other populations is not possible. Additionally, SP visits were not audiotaped. Audiotaping can improve the reliability of manually documented data and result in more accurate assessments [28].

Some have expressed ethical concerns in relation to the SPs method, as pharmacists do not give consent to participate. However, others have argued that it can be a robust method for assessing practice, and may be justified in the wider public interest [16].

The operational classification we used to assess counselling was originally used within the context of prescription medicines [18]. In such cases, a patient should already have consulted with a medical practitioner and pharmacists’ questioning serves the purpose of meeting specific patient needs only. However, in the present study two scenarios used OTC medications. For OTC medications, pharmacists’ questioning serves a broader purpose. It should not be limited to previous use, allergies, use of other medicines, knowledge of indications, or dosing instructions, but should include an assessment of other aspects, namely nature of the symptoms, treatment duration, and current conditions. The latter were not investigated in this study.

The current study assessed the counselling process only for situations where patients specifically request a medication by name. Other situations, such as counselling for patients with prescriptions and for patients approaching pharmacists for treatment (i.e. explaining their symptoms to pharmacists, who then provide them with the medications), were not investigated. Therefore, our findings cannot be extrapolated to such situations.

## Conclusion

The present study highlights the current deficiencies in appropriate dispensing practices and medication counselling at community pharmacies in Saudi Arabia. Policy makers, stakeholders, and researchers should collaborate to design interventions to improve the current dispensing practices at community pharmacies across the country.

## Additional file

Additional file 1:
**Questionnaire of patient counselling in the community pharmacy.** (PDF 294 kb)

## References

[1] Nkansah N, Mostovetsky O, Yu C, Chheng T, Beney J, Bond CM (2010). Effect of outpatient pharmacists’ non-dispensing roles on patient outcomes and prescribing patterns. Cochrane Database Syst Rev.

[2] A Call to Action (2007). Preventing U.S. citizens from inappropriate medication use. a white paper on medication safety in the U.S. and the role of community pharmacists.

[3] American Society of Health-System Pharmacists (1997). ASHP guidelines on pharmacist-conducted patient education and counseling. Am J Health Syst Pharm.

[4] Okumura LM, Rotta I, Correr CJ (2014). Assessment of pharmacist-led patient counseling in randomized controlled trials: a systematic review. Int J Clin Pharm.

[5] Eickhoff C, Hämmerlein A, Griese N, Schulz M (2012). Nature and frequency of drug-related problems in self-medication (over-the-counter drugs) in daily community pharmacy practice in Germany. Pharmacoepidemiol Drug Saf.

[6] Nicolas A, Eickhoff C, Griese N, Schulz M (2013). Drug-related problems in prescribed medicines in Germany at the time of dispensing. Int J Clin Pharm.

[7] Puspitasari HP, Aslani P, Krass I (2009). A review of counseling practice on prescription medicines in community pharmacies. Res Social Adm Pharm.

[8] Ministry of Health. Health statistical yearbook (2014). http://www.moh.gov.sa/Ministry/Statistics/Book/Pages/default.aspx. Accessed December 2015.

[9] The Saudi Food and Drug Authority. Executive Roles for Institutions and Pharmaceutical Products Law. http://www.sfda.gov.sa/AR/DRUG/DRUG_REG/Pages/drug_reg.aspx. Accessed December 2015.

[10] Alghanim SA (2011). Self-medication practice among patients in a public health care system. East Mediterr Health J.

[11] Easton K, Morgan T, Williamson M (2009). Medication safety in the community: A review of the literature.

[12] Bin Abdulhak A, Al Tannir M, Almansor M, Almohaya M, Onazi A, Marei M (2011). Non prescribed sale of antibiotics in Riyadh, Saudi Arabia: a cross sectional study. BMC Public Health.

[13] Al-Mohamadi A, Badr A, Bin Mahfouz L, Samargandi D, Al AA (2013). Dispensing medications without prescription at Saudi community pharmacy: Extent and perception. Saudi Pharm J.

[14] Alte D, Weitschies W, Ritter CA (2007). Evaluation of consultation in community pharmacies with mystery shoppers. Ann Pharmacother.

[15] Watson MC, Bond CM, Grimshaw JM, Johnston M (2006). Factors predicting the guideline compliant supply (or non- supply) of non-prescription medicines in the community pharmacy setting. Qual Saf Health Car.

[16] Weiss MC, Booth A, Jones B, Ramjeet S, Wong E (2010). Use of simulated patients to assess the clinical and communication skills of community pharmacists. Pharm World Sci.

[17] Gokcekus L, Toklu HZ, Demirdamar R, Gumusel B (2012). Dispensing practice in the community pharmacies in the Turkish Republic of Northern Cyprus. Int J Clin Pharm.

[18] Tully MP, Beckman-Gyllenstrand A, Bernsten CB (2011). Factors predicting poor counselling about prescription medicines in Swedish community pharmacies. Patient Educ Couns.

[19] Watson MC, Norris P, Granas AG (2006). A systematic review of the use of simulated patients and pharmacy practice research. Int J Pharm Pract.

[20] Micromedex® Healthcare Series, Thomson Micromedex, Greenwood Village, Colorado (Edition expires [12/2015]).

[21] Berger K, Eickhoff C, Schulz M (2005). Counselling quality in community pharmacies: Implementation of the pseudo customer methodology in Germany. J Clin Pharm Ther.

[22] Schommer JC, Wiederholt JB (1997). The association of prescription status, patient age, patient gender, and patient question asking behavior with the content of pharmacist-patient communication. Pharm Res.

[23] Morris CJ, Cantrill JA, Weiss MC (1997). “One simple question should be enough”: Consumers’ perceptions of pharmacy protocols. Int J Pharm Pract.

[24] Nair K, Dolovich L, Cassels A, McCormack J, Levine M, Gray J (2002). What patients want to know about their medications: Focus group study of patient and clinician perspectives. Can Fam Physician.

[25] Svarstad BL, Bultman DC, Mount JK (2004). Patient counseling provided in community pharmacies: effects of state regulation, pharmacist age, and busyness. J Am Pharm Assoc.

[26] The Saudi Food and Drug Authority. Registered drugs and herbal product list http://www.sfda.gov.sa/ar/drug/search/Pages/default.aspx. Accessed December 2015.

[27] Kansanaho H, Pietilla K, Airaksinan M (2003). Can a long term continuing education course in patient counseling promote a change in the practice of Finnish Community Pharmacists?. Int J Pharm Pract.

[28] Werner JB, Benrimoj SI (2008). Audio taping simulated patient encounters in community pharmacy to enhance the reliability of assessments. Am J Pharm Educ.

