# Supplementary material for: Counselling practices in community pharmacies in Riyadh, Saudi Arabia: a cross-sectional study
Source: BMC Health Serv Res. 2015 Dec 15;15:557. doi: 10.1186/s12913-015-1220-6 (PMC4678714; doi:10.1186/s12913-015-1220-6)
Supplement: Additional file 1: — Questionnaire of patient counselling in the community pharmacy. (PDF 294 kb) [file 12913_2015_1220_MOESM1_ESM.pdf]

Dear Pharmacists Code ☐

We are trying to understand the process of patient counseling in community pharmacies. We greatly appreciate you help in filling this questionnaire . **Dr. Alaqeel S and Dr. Abanmy N. College of pharmacy, King Saud University.**

1. **Age**:

☐ 20-30 years old ☐ 31-40 years old ☐ 41-50 years old ☐ > 50 years old

2. **Degree:**

☐ BSc. ☐ PharmD ☐ Other: ………………………….(Please specify)

3. **Nationality:**

☐ Saudi ☐ Non- Saudi ………………… (Please specify)

4. **How long have you been working as a community pharmacist?**

☐ Inside Saudi Arabia ………… years ☐ Outside Saudi Arabia ………. Years

5. **How long do you spend in the pharmacy/premise in a day?**

☐ Less than 8 hours ☐ From 8-12 hours ☐ More than 12 hours

6. **Do you have assistant employee in the pharmacy?**

☐ No

☐ Yes

if yes, are they ?

☐ **Pharmacists** ☐ **Pharmacy technicians** ☐ **Others**

7. **How long do you spend approximately for dispensing a single drug?** ………. **seconds**

8. **Approximately, How many patients visit your pharmacy per day?**

WITH a prescription

☐ **0-10** ☐ **11-20** ☐ **21-30** ☐ **31-40** ☐ **More than 40**

WITHOUT a prescription

☐ **0-10** ☐ **11-20** ☐ **21-30** ☐ **31-40** ☐ **More than 40**

9. **How often do you inform the patients about the following issues when dispensing a medication....**

	Always	Usually	Often	Sometimes	Never
**The purpose of medication or diagnosis**	5	4	3	2	1
**Dosing of the drugs**	5	4	3	2	1
**Information on how to use the medication and its application**	5	4	3	2	1
**Medication to be taken with food or on an empty stomach**	5	4	3	2	1
**Duration of use**	5	4	3	2	1
**Possible side effects**					
**Drug interactions**	5	4	3	2	1
**Food interactions**	5	4	3	2	1
**Importance of compliance**	5	4	3	2	1
**Storage conditions**	5	4	3	2	1
**Availability of generic medication**	5	4	3	2	1

10. **To which degree you agree to the following statements about your counseling ......**

	Strongly Agree	Agree	Unsure	Disagree	Strongly Disagree
**Patients are comfortable in consulting me about their medication/medical condition**	5	4	3	2	1
**I use all the opportunities to clarify patients understanding of my counseling**	5	4	3	2	1
**Patients understand the information I provide them**	5	4	3	2	1
**I confirm and clarify the understanding of the patient**	5	4	3	2	1
**I am satisfied with my counseling practice**	5	4	3	2	1

11. **To which degree you agree that the following are barriers for providing patient counseling in community pharmacies .....**

	Strongly Agree	Agree	Unsure	Disagree	Strongly Disagree
**Pharmacists have limited drug information resources**	**5**	**4**	**3**	**2**	**1**
**Pharmacists are too busy**	**5**	**4**	**3**	**2**	**1**
**Pharmacists do not have the patient medical history**	**5**	**4**	**3**	**2**	**1**
**Pharmacists lack confidence in their knowledge**	**5**	**4**	**3**	**2**	**1**

Any other barriers? ....

THANK YOU FOR YOUR TIME
